# Philanthropic Investment in Equity: Cultivating Grass Roots Leaders for the Equitable Revitalization of Marginalized Communities

**DOI:** 10.1007/s42413-021-00159-x

**Published:** 2022-02-23

**Authors:** Jason Reece, Bernadette Hanlon, Ryan Edwards

**Affiliations:** 1 City & Regional Planning, Knowlton School, Ohio State University, Columbus, OH USA; 2 Collective Impact & Student Success, The United Way of Central Ohio, Columbus, OH USA

**Keywords:** Engagement, Leadership, Community Development, Gentrification, Social Capital

## Abstract

Community development must include deeper investment to foster a pipeline of community leaders to support equitable redevelopment practice in marginalized communities under threat of gentrification in the city. We argue that philanthropy is critical to develop this pipeline, particularly in the era of the neoliberal city. The following case study analyzes efforts to develop place-based grass roots leadership in marginalized neighborhoods of Columbus, Ohio. The United Way of Central Ohio, through their Neighborhood Leadership Academy (NLA) program, has partnered with community organizations to develop multiple cohorts of grass roots neighborhood leaders over several years within three specific neighborhoods. Our case identifies how philanthropic investment into a grass roots leadership development model centered on equity has impacted policy outcomes, built bridging social capital and spurred successful activism. Our case illustrates a potential model for building social infrastructure through philanthropic investment to buttress potentially disruptive neighborhood change. In the era of the neoliberal city, neighborhoods can no longer rely upon federal funding, leaving redeveloping neighborhoods particularly vulnerable to market driven gentrification and displacement. In this void of resources, philanthropic efforts to support robust grass roots leadership is the last remaining defense against widespread displacement and the primary asset to support equitable development practices.

## Introduction

The communicative model and its emphasis on citizen engagement has largely become a vehicle for middle class interests in public sector decision making (Fainstein, 2011). We are at a crossroads in terms of rethinking approaches to genuine community participation, engagement and empowerment, particularly in meeting the needs of marginalized communities threatened by gentrification in the city. Contemporary planners seeking to support robust engagement within marginalized neighborhoods face community barriers pertaining to *trauma, trust and time* (Mullainathan & Shafir, 2013; Uslaner & Brown, 2003; Weinstein et al., 2014). These factors are especially acute for marginalized communities sensitive to gentrification processes and the prospect of displacement (powell & Spencer, 2002).

Community development must include deeper investment to foster a pipeline of community leaders to support equitable redevelopment practice in marginalized communities under threat of gentrification in the city. We argue that philanthropy is critical to develop this pipeline, particularly in the era of the neoliberal city. In this paper, we analyze efforts to develop place-based grass roots leadership in marginalized neighborhoods experiencing redevelopment in Columbus, Ohio. The United Way of Central Ohio, through their Neighborhood Leadership Academy (NLA) program, has partnered with community organizations to develop multiple cohorts of grass roots neighborhood leaders over several years within three specific neighborhoods.

The growth machine politics of the Columbus region can frustrate local community activism (Smola & Ferenchik, 2019; Webb, 2013). This case study focuses on the development of a multi-year intervention in several neighborhoods undergoing market driven redevelopment in the city of Columbus. The NLA program emerged as a philanthropic partnership with community organizations to build a stronger pipeline of leaders to support equitable development. As a result, more than 100 leaders have emerged to create a new layer of social capital and activism for these neighborhoods which are under significant redevelopment pressure. Our case study includes interviews, surveys and participant observation documenting the evolution of this grass roots leadership development effort over the past five years. Our case identifies the way philanthropic investment into a grass roots leadership model has impacted policy decisions, built bridging social capital and spurred successful activism. Our case illustrates a potential model for building social infrastructure through philanthropic investment to buttress potentially disruptive neighborhood change.

## Philanthropy, Civic Engagement and the Neoliberal City

In this case study, we bring together different strands of scholarly literature relevant to our understanding of the contribution philanthropic organizations make to equitable community development in marginalized communities in the city. The first area of previous research relates to the increasing relevance of philanthropic organizations in community and economic development broadly.

Philanthropic foundations and organizations are now well-recognized as key stakeholders in the revitalization and economic growth of cities (Martinez-Cosio & Rabinowitz Bussell, 2013). These philanthropic initiatives are primarily place based and have been categorized as attempting to utilize spatial approach to addressing interconnected social challenges while building community capacity and aligning various stakeholders (Murdoch et al., 2007). Some scholars question if these kinds of organizations adequately distribute resources to those most in need (Díaz & Shaw, 2002, Eikenberry, 2006) or if they can foster real social change that is beneficial to marginalized groups and neighborhoods (Scott et al., 2020; Nickel & Eikenberry, 2009). Pill’s (2019) case analysis of philanthropic place-based revitalization efforts in Baltimore and Cleveland found efforts primarily aligned with exiting neoliberal policy agenda of more powerful political stakeholders and were less successful in promoting the agency of neighborhood residents. Scholars have also long recognized the power imbalance between philanthropic institutions and community stakeholders in redevelopment initiatives and the limited racial, ethnic and economic diversity in philanthropic leadership (Azevedo et al., 2021; Barkan, 2013).

Others, in contrast, suggest that philanthropic organizations have a diverse range of economic development strategies that encompass social equity components (Giloth, 2019), and that philanthropic groups, more so than local governments, can be flexible and innovative, take risks and collaborate on initiatives to bring about significant social transformations and change. Recently, philanthropic coalitions have emerged to engage in significant economic development initiatives in the city. One prominent example is the Living Cities, a collaboration of large foundations and financial institutions that aim to improve the lives of low-income residents in some 40 cities across the United States (Giloth, 2019). Doctor’s (2014) case analysis of philanthropic investment to support neighborhood revitalization in East Oakland, CA to have produced more equitable outcomes by centering the initiative on the needs of the community by integrating active listening, acknowledging power imbalances, practicing cultural humility and prioritizing the voices of community stakeholders.

In conjunction with these kinds of large-scale collaborations, the work of individual philanthropic organizations can be significant in the realm of community capacity building. A recent trend in the world of philanthropy is to support community-led development strategies, and for philanthropic organizations to seek ways to gain community buy-in for the purposes of community planning and development. In this role, philanthropic organizations can be instrumental as leaders in local community development efforts. Philanthropic organizations, using the tools of community assessment, can serve to be facilitative leaders themselves, and they can promote community dialogue and collective action (Shier & Handy, 2015). Although Bonds et al.’s (2015) research has found that non profit and philanthropic organizations that seek to foster grass roots leadership in neighborhood improvement too often are led by a “colorblind” lens of community development, which can create more conflict and further marginalize communities of color.

In this paper, we focus on the efforts of a philanthropic organization to build neighborhood capacity by developing leaders within the community. As mentioned, we examine the United Way of Central Ohio’s Neighborhood Leadership Academy as a tool for the development of grassroots leadership in different marginalized communities in Columbus Ohio. We argue that the development of grassroots leaders in these communities is necessary to offset threats of neighborhood gentrification. They act as primary assets to support equitable development in city neighborhoods undergoing rapid change.

This brings us to our second strand of literature, the role of community leadership in community development efforts and community wellbeing. Community, in this context, is a geographic location as well as a space of shared interests and experiences (Walker, 2008). Community leadership, as we define it in this paper, occurs at the neighborhood scale, and community leaders also include non-elected and informal leaders. Grass roots leadership can instigate changes within their neighborhood through communication and cooperation with larger stakeholders. Mundell et al. (2015) find that grassroots leadership development and collaboration is more effective than top down advocacy in preventing gentrification-based displacement.

We must recognize leadership development as not just skill development but relational development and enhanced connectedness. Social connectedness to both place and people is associated with increased wellbeing, life satisfaction, flourishing and hope (Munoz et al., 2020). Cloutier, Ehlenz and Afinowich find in addition to material resources, infrastructure and amenities that ‘purpose, place, and relation’ are critical to community wellbeing (Cloutier et al., 2019). Ideally relations should be across a wide spectrum of difference and be “reciprocal and empathetic” and in practice emphasize “being and interacting” within the community (Cloutier et al., 2019).

## Case Methods

We utilized a single case study approach to understand the impacts and influence of the NLA on resident empowerment, skill development, social capital formation and impacts on policy and community initiatives. More specifically we sought to understand how the NLA impacted alumni, influenced relationships and affected decision making or community initiatives. More broadly, we sought to utilize the NLA case to better understand the limitations and potential of sustained equity focused grass roots leadership development on countering the potential detrimental effects of gentrification and displacement.

We utilized Birch’s (2012) review of case study classification to align the case design with best practices in case analysis for planning practice. Our case is action oriented (emphasizing implications for practice), focused on the impact on a specific population (marginalized communities within neighborhoods facing redevelopment pressure) and reevaluating a substantive issue in scholarship (countering gentrification pressures). Informed by Yin’s (2014) categorical definitions of case studies, our case is both exploratory (seeking to understand how leadership development impacts individuals and communities) and descriptive (seeking to describe how enhanced social capital can impact neighborhood development processes). We triangulated several sources of data to develop the case study including participant observation, focus groups, surveys and interviews. Survey questions and semi-structured interview questions are provided in Appendix. In addition to data triangulation, our methods pulled from additional practices to enhance internal validity (Yazan, 2015) these additional techniques are described in the limitations section below.

### Participant Observation

Participant observation was conducted over the course of four years (2015–2019). Participant observation included early planning activities designing the structure of the three NLA programs, participation in NLA events and observation of NLA alumni in community meetings and working groups. Approximately fifty hours of time was spent participating and observing for the case analysis over a four-year time span.

### Focus Groups

In collaboration with graduate students participating in a city planning studio course, we held three focus groups with both NLA alumni, residents in the Linden neighborhood and NLA program directors. Semi structured questions were utilized to understand the experience of both alumni, residents and program directors/staff. The first two focus groups focused on residents in the Linden community (where the NLA was going to be launched) and the program alumni from both the City wide and South Side NLA. The third focus group specifically focused on the experience of NLA program directors from Linden, the Near East Side and the South Side. All focus groups occurred in the fall and winter of 2017/2018. Approximately three dozen participants in total attending the focus group meetings.

### NLA Alumni Survey

A Qualtrics survey of NLA alumni occurred in the spring of 2019. The survey was distributed to 100 alumni of the various NLA programs and received a 17% response rate. Due to the low survey response rate, a series of alumni and program director interviews were conducted to provide additional data.

### Alumni and Program Director/Staff Interviews

In the summer and autumn of 2020, zoom based semi-structured interviews were held with six of the South Side NLA alumni and one NLA program director and NLA program staff member. The NLA program director and staff member were both involved in managing and providing curriculum for the NLA on the South Side.

### Analysis

Due to the low survey response rate, survey data was only summarized as descriptive data. Qualitative data collected in surveys, focus groups and interviews were transcribed and analyzed through both inductive and deductive coding. Field notes from all participant observations were also analyzed to inform the analysis. Additionally, program reports and other administrative documents were reviewed to support the case study.

### Limitations

Our case analysis has limitations, the limited number of focus groups (2), and limited sample of participants included in interviews (8) and surveys (17) is a limiting factor. To strengthen the validity of our findings we have we utilized best practices in case methods (triangulation, prolonged engagement, persistent observation and extensive member checking) (Denzin, 1978, Lincoln & Yuba, 1985, 1986, Yazan, 2015). Our approach at triangulation emphasized data source triangulation, methods triangulation and analyst triangulation (Yin, 2013). These strategies have strengthened the internal validity of our case analysis, we caution that the case has limited external validity (or generalizability to other community settings). While not generalizable, the case analysis does provide a basis for further research in other community settings.

## Case Background & Context

Unlike other midwestern regions, the city of Columbus has experienced substantial population growth in recent decades, growing from 540,000 residents in 1970 to more than 900,000 residents in 2020. The metropolitan region’s population would double in size during this time frame (U.S. Census Bureau, 2020). In the past two decades Columbus has experienced growing inequality as the region’s resurgence masked a growth in the population in poverty and economic segregation (Price, 2015). As a growing city and region, the city’s urban core neighborhoods that had lost population in the preceding decades and were deeply impacted by the 2008 foreclosure crisis have experienced a resurgence.

**Fig. 1:**
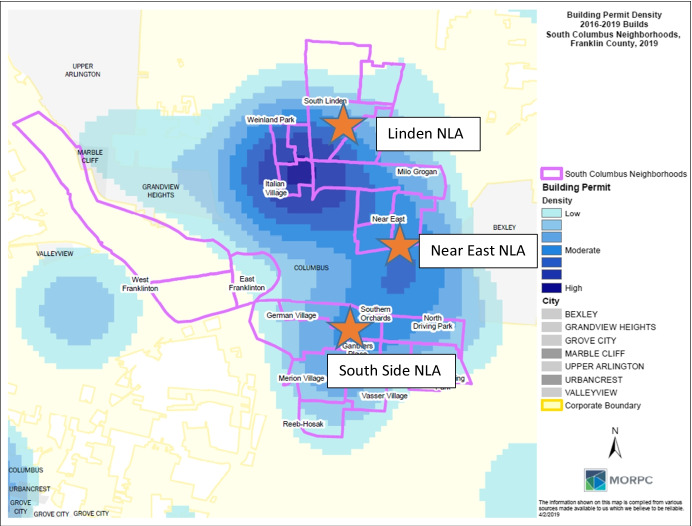
NLA locations and development activities in the core of the City of Columbus Ohio

As seen in Fig. 1, recent development activity has grown tremendously in urban core neighborhoods. The development of the local NLA’s in the South Side, Near East Side and Linden community coincides with increasing development pressure near and within the neighborhoods. The nexus of growing inequality and urban redevelopment has raised fears of displacement fueled by gentrification in the city’s core urban neighborhoods. Columbus’s identity as embracing “growth machine” politics and neoliberal public private partnerships has contributed to concerns about political disengagement and marginalization in neighborhoods in which developers have significant political power in driving redevelopment (Smola & Ferenchik, 2019; Webb, 2013). These dynamics and the city’s political history were directly referenced in the founding of the academies, as one former NLA program director stated *“we (planners) have created the apathy (among residents) because of the legacy of engagement which is after the fact.”*

Scholars have recognized that community demands for equity in the context of the growth machine are more likely to be successful in places with more economic growth (Cain, 2014). Community members have more leverage in demanding equitable development in neighborhoods where developers have a higher potential for profit. From this perspective, the regional market conditions of Columbus and the strong neighborhood real estate conditions, in neighborhoods targeted by the NLA, create more potential for leveraging community voice.

The NLA was first launched as a city-wide leadership development program by the United Way of Central Ohio in 2012. The citywide program differs in many ways from the ‘hyper local’ neighborhood focused academies that we study for this case. The city-wide program was primarily focused on developing leaders who would fill more structured roles as volunteers, board or committee members or those working in the nonprofit community. The city-wide program mirrored more traditional neighborhood leadership programs commonly found in cities across the nation.

The city-wide program curriculum overlapped with the local neighborhood academies in some aspects, but the local NLA academies focused more on grass roots and informal leadership development, with curriculums that were structured around the unique characteristics, assets and issues relevant to each neighborhood. The local NLA programs were led by a local community organization in collaboration with the United Way of Central Ohio and often involved partnership with Ohio State University. Local NLA’s recruit from local stakeholders who live, work, learn or worship in the neighborhood. A selective recruiting process produces cohorts of approximately ten to twenty participants who take part in nine months of learning, relationship building and direct community work. Cohorts work on several community projects created collectively within the cohort. The local academies were inspired by a goal of creating a large cohort of emerging leaders in each community over a multi-year time span.

The local neighborhood development academies first launched in the South Side community in 2015 as a collaborative partnership between the neighborhood’s CDC (Community Development for All People or CD4AP), Nationwide Childrens Hospital (the primary anchor institution in the neighborhood), the United Way of Central Ohio and the Kirwan Institute at Ohio State University. The Near East side neighborhood academy was developed in 2017 and the Linden neighborhood academy was launched in 2018. The South Side academy is entering its sixth year of programming and has more than 75 alumni from the first five years of the program. The Near East Side and Linden academies ran for two years but have suspended operation.

### Curriculum: Assets Based and Committed to Equity

The local NLA curriculums placed emphasis on asset-based models of community development and commitment to supporting equity, inclusion and diversity in the neighborhoods. The focus on assets, equity and inclusion is centered in all aspects of the program design, from recruiting materials, to class-based curriculum and engagement within the community. Program directors noted that communicating the “values’ of the academy was critical in helping recruit applicants who were committed to those values. As one former academy director described.

*“People self-select in or out based on what they see in the application, (this is) the* *intentionality of the program design, and this gets further enforced through the* *curriculum. What is taught, how it’s taught and who is teaching it, that is not just left to* *chance. (We) define in the wording of the application, what is the culture you are trying* *to promote, cast the vision and values in the application early on, this is about change.”*

For example, the themes equity & inclusion are very evident in descriptive text utilized to market the South Side academy: *“We seek participants that aspire toward a safe, opportunity-rich South Side that maximizes diversity, builds relationships, secures and advances current residents, all while maintaining the unique fabric of the community”*(CD4AP, 2020).

Program curriculum focuses explicitly on issues of racial bias and the ways structural racism has impacted the neighborhoods historically or in the present. As described by an NLA curriculum provider: *“(The curriculum) particularly is strong in topics related to race and ethnicity, it is in a way that it won't turn anybody off won't take the conversation where it's not healthy. The (curriculum) gives people the opportunity to approach new knowledge and be self-reflective about themselves, how they've seen the community, how they have seen community problems, and see it at a different light.”*

Philosophies of asset-based development are continually structured into the application materials, activities and programming. As described by an NLA program director. *"We don’t ask on the application about needs, we ask about assets, What are your goals, hopes, aspiration, inspiration, skills you bring?”* Asset mapping within the context of the neighborhood is also essential to helping participants shift their perspective in how they view the community’s resources and opportunities. As described by an NLA program director. *“The asset mapping is very important – because people don’t think about or see the assets in their community, key to changing the lens of the individual, very important to use asset based language in the program, what are the talents, gifts, treasures that exist in each neighborhood.”*

Asset based perspectives also places emphasis on the community’s population diversity as its primary asset. As described by an NLA program director: “*We’re driving home the notion that (being) asset based will always get you further and that our people will always be our greatest asset and never a deficit.”*

### Diversity & Group Heterogeneity

Substantial deliberation is given to the applicant review process, specifically with the intention of producing a highly heterogenous class cohort each year, which represents an extensive representation of difference within the neighborhood. As described by the South Side NLA director. *“The South Side neighborhood Leadership Academy, probably not unlike the other ones is incredibly diverse across race, class and education background. It's always multi-racial, it's the full income spectrum, folks that are making six figures, folks that are making no figures. We had multiple people with PhDs. We've got people that don't have GED’S and so it is the full range.”*

NLA directors emphasize the importance of balancing different skill sets and life experiences in the class cohort, with a specific emphasis on creating a learning environment that de-centers the knowledge of participants who are more privileged and formally educated and trained. As described by the South Side NLA director, this means creating a space of restraint, listening and self-reflection for individuals who are often very assertive in their leadership style. Growing cultural humility among more privileged participants is a direct goal, as described by the South Side NLA director: *“Particularly for our middle- and upper-class folks, particularly for our white folks, the biggest lesson that I'm trying to (assure) that most of them get by the end, is they don't have all the answers. In fact, they're the ones with much to learn.”*

## Analysis

Our case analysis focused on understanding several key outcomes from the NLA. In regard to impacts on alumni we explored skills development, participant engagement and shifts in perspective. Social capital formation identified the extent of relationship building and the types of social capital developed (bonding or bridging) among alumni. Survey, focus group and interview data indicate NLA alumni feel the program has been beneficial in strengthening their skills, relationships and political connections and deepening their perspectives on the neighborhood and issues of redevelopment. Alumni are highly engaged and politically active. Alumni have more mixed perspectives on the long-term potential of the NLA to counter the potential for displacement in the neighborhood.

### Skill and Network Development

Alumni in surveys, interviews and focus groups were asked to identify what skill development was most important from their time in the academy. In surveys participants ranked the following as the most important skills learned in the NLA.

1. Community engagement techniques
2. Recognition of community assets and local neighborhood knowledge
3. Personal development
4. Fundraising/grant writing
5. Networking development/coalition building
6. Community development strategies/tools (equitable development practices)

Alumni in focus groups and interviews generally identified similar skill development. Focus group participants primarily emphasized their engagement with community assets, particularly places they had never been exposed to previously. This identification of new assets deepened their pride in the community. In addition to community assets, interview participants primarily discussed fundraising and grant writing training as important new skills learned.

Both focus group and interview participants focused extensively on how the NLA expanded their personal networks, community partnerships and political connections. The average NLA alumni was still connected or collaborating with at least four other NLA alumni. Participants particularly focused on leveraging their new connections and social networks to support community initiatives or solve community challenges. As described by NLA alumni.

*“I just recently…launched a new training program called the bridge to self-sufficiency and I got an opportunity again through all these connections that have been built through my relationships that launched with NLA. So now I've launched an actual program that I'm trying to get funding for.”*

*“I have learned how to effectively create a change in my community by reaching out to stakeholders, community leader, and resident to find the best way to get the needs meet as a whole.”*

*“I think the biggest thing for me is that that not only the program, but then the people that they brought in to talk to us and participate or like, you know, we might (City Council president) Shannon Hardin and we met (state) house representatives and actually having access to people like that and feeling like you're being heard and like if you want. Like I said, if you want to be involved. If you wanted to do something positive, like you have all of these inroads so I feel incredibly connected to Columbus.”*

### Shifts in Perspectives: Embracing and Asset Based Lens

As previously discussed, participants routinely identified the program as effective in teaching them about local neighborhood assets that they were unaware of. In interviews, alumni discussed that their views on assets extended beyond just focusing on neighborhood assets but represented a more robust embrace of an asset based perspective for personal development and problem solving. As articulated by an NLA alumni.

*“The one big thing I took away from the NLA was the whole concept of looking at a challenge and seeing it as an opportunity, and just kind of like imbuing that idea across everything that we did. You know that that all you really need is you and your resourcefulness. You can bring that mindset to whatever the next step is and whatever it is you're doing or whatever process you're managing or leading. (The NLA) really emphasized spending time with each individual person on (identifying) their own assets, and being able to name what they are, and recognize them as assets.”*

### Building Perspective and Relationships Across Lines of Difference-

Bridging social capital, is social capital formation developed between lines of difference. Participants routinely identified growth in deepening their personal relationships and perspective across various lines of difference as a key benefit of the program. This relationship building was essential in broadening their horizons through exposure to places and people outside of their personal experiences in the neighborhood. In many cases, more privileged and economically affluent participants discussed how this cross-cultural learning was critical as a learning experience to reshape their views of the community. As one NLA alumni articulated.

*“Doing the, the leadership academy gave me an opportunity to sort of break that bubble and get to know people who are living a little bit grittier and have people who've grown up, you know, in the heart of the South Side and seeing, you know, seeing things and been a part of life here for a long time. So a lot of the people who live in my neighborhood now are new. I mean, we still have old timers. But there's a lot of newbies like us, so I do feel more connected because of that knowing people who are sprinkled all over the area and hearing their stories.”*

Bridging social capital development was also important to shift existing perspectives on neighborhood change and development. Alumni commonly referenced their lack of depth and understanding on the impacts of gentrification, particularly understanding the impacts to longtime residents and economically marginalized community members. The shift in perspective around issues of displacement and neighborhood change was routinely referenced in interviews by alumni.

*“Oh, I (gained) an enlightenment of (the process of) displacement.”*

*“White middle class folks will explicitly say. ‘I know I'm part of these market forces. How do I fix it?’ Then the folks (that have lived) here for the long term are pretty clear and explicit about how much the neighborhood's been changing and the effects that's had on their lives, the effects that it's had on their neighbors that have been pushed out there. So really, from day one of class that's top of mind for people. And then we dig into, you know, what do we do about it, that sort of thing.”*

*“It changed my mindset because like I said when I first came down here…I just thought property values are going to go up. Awesome! And now (I understand) at what cost, it's good to see the neighborhood improve. We've definitely had some issues with the drugs, prostitution and all of the joys that come with that but (the NLA’s) given me insight into how pushing that further down the road to the next neighborhood is not the solution.”*

### Political Engagement, Influence on Decision Making and Community Initiatives-

The South Side NLA has been the longest running academy and has the largest cohort of alumni active in the neighborhood. Our case analysis finds that the NLA alumni on the South Side have remained politically engaged, had direct impact on policy/decision making, advocacy and launching new community initiatives. We did not have enough data to accurately gauge the long-term impacts on policy and political outcomes in the Near East Side and Linden academy.

Alumni have also embedded themselves into local civic associations and boards and are highly engaged. In surveys two thirds of alumni indicated they were actively involved in at least two ongoing community programs, initiatives or organizations. Surveys indicated that 66% of alumni work 4 or more hours a week on community issues and 40% work more than 10 h a week. These have included more informal positions leading grass roots initiatives and more formal positions with policy and decision-making entities in the neighborhood. As described by NLA program directors and alumni.

*“I just became a Far South Area Commissioner (the local governing body for Columbus neighborhoods). So now I'm actually like civically involved as well and on a couple of different boards.”*

*“We do find there's a significant number of our alumni that are in leadership and their civic associations or their area commissions, after having gone through the program. And I wouldn't say that that's the driving motivator (for joining the NLA).”*

Alumni have played a direct role in policy/decision making and joined successful advocacy efforts to dispute inequitable policy decisions. These efforts have ranged from building support for affordable housing development, stopping a disruption in neighborhood transit services and fighting the closure of schools in the neighborhood. Alumni have also successfully transitioned some of the NLA community projects into more robust community programs. For example, the ID program, a program focused on removing barriers for obtaining state identification to assist marginalized residents in accessing benefits and voting, expanded and received direct support from funders in the community. As described by an alumnus.

*“Well, I'm really excited because our project actually turned into a full-fledged program that was funded by the city. Our project was helping folks who did not have proper ID to acquire it because without state ID, you can't get a job. Yeah. You can't access resources and benefits that you may be entitled to or need so we thought that it was a real critical area where we had an opportunity to make a difference. So, with our funds we actually connected with the local Bureau of Motor Vehicles and identified other obstacles to people getting their ID. It's not just about having eight bucks to go in and get it. You gotta have a birth certificate. You got to have proof of residence and all these other things. So, we are investigating what was required and realize that maybe transportation was an obstacle to somebody being able to even get to the Social Security Office nowadays.”*

### Can NLA Alumni Help Prevent Displacement?

NLA alumni were more mixed in their perspectives on grassroots efforts to counter displacement produced by gentrification. While alumni deepened their understanding of the consequences of displacement and who was most vulnerable, they were less sure about how effectively they can prevent it in the long term. While 2 out of 3 survey respondents felt their work would help counter gentrification and displacement harming marginalized community residents, interview participants were mixed in their response to this question. Alumni who were optimistic in this regard emphasized the ongoing work of the local CDC (Community Development for All People) and hopes for more balanced growth.

*“Organizations like the Church For All People and this academy and all of the things that they're involved in like knowing that their groups active and pushing for the right things and are actually making strides. That makes me feel (positive, but) I don't think we're going to get out of it unscathed. I certainly think that.”*

*“Nobody likes change but (can we) help balance and way that change affects the future of the community in a positive way, it’s…something that I'm really honored to be a part of. And again, I think it all started with the NLA.”*

More pessimistic alumni expressed a need for a deeper skill set to counter gentrification, the vulnerability of lower income renters and skepticism about being able to counter the political influence of developers.

*“And (in the end) money will talk and eventually this developer will be able to get what he wants.”*

*“(We need) …better training on fighting capitalism and gentrification.”*

*“What I feel like we're missing those potentially marginalized renters, who should have a voice, but maybe don't know that they do or aren't engaged enough to even be asked…my focus is, how do we get more involvement.”*

*“I don't know, I think that some (displacement) is inevitably going to happen. That's going to be inevitable.”*

NLA program directors present a more positive, long term and macro view of the potential of the program in countering displacement and supporting a diverse opportunity rich neighborhood. The South Side program director referenced the cumulative effect of five years of NLA alumni active in the community, with many in decision making roles.

*“One of our big goals for the south side neighborhood Leadership Academy is this notion that we've got now got close to 75 people running around the south side that have sort of drunk the Kool Aid around valuing a diverse mixed income opportunity rich community…they are the ones having the front porch conversations, they are, frankly, the one sitting in civics (civic associations) and Area Commission leadership.”*

The South Side NLA has utilized NLA members to engage and counter Not In My Backyard (NIMBY) resistance to affordable housing in the community. The program anticipates strategically leveraging the support of NLA alumni as the local CDC and other community partners seek to expand the stock of affordable housing in the community.

*“We've become very intrigued about how we leverage the academy, particularly given that the (alumni) are on Commission's or chairing zoning committees. So that when the next multi-unit affordable housing goes up for approval, we flip the nimby’s into seeing that is value added. There was going to be multi-unit affordable housing LIHTC development (that) was trying to get through the area commission and just meeting with tons of opposition and NIMBY’ism. They (the developer) hadn't necessarily done their due diligence in getting community buy in ahead of time. But we were fairly successful in sort of beating the bushes for our alumni that lived in that area to say, here's the conversation that is happening. Would you be willing to get involved to provide another perspective?”*

## Discussion & Conclusion

Leadership development is more than skills development but relational development and shifts in perspective. We contend that grassroots leadership development programs, if equity centered and sustainable can build political power, enhance activism, spark grassroots initiatives and foster bridging social capital to counter inequities produced by gentrification in neighborhoods experiencing reinvestment. Increasing awareness and anxiety among “newcomers” who feel they may be causing unjust outcomes in neighborhoods under transition create an opportunity to fuel relationship building and activism across lines of difference. The development of bridging social capital is part of equitable neighborhood development that should be emphasized. The NLA experience also presents an application in practice of centering equity and decentering Whiteness and privilege in neighborhood contexts.

### The Importance of Programs Centered on Values of Equity & Inclusion-

Equity planning has long contended that value neutrality in planning is problematic, and an open and robust embrace of equity as a value is essential (Davidoff, 1965). In the context of cultivating grass roots leadership development, values must lead the structure, curriculum and recruiting of programs. The NLA has been very effective (particularly the South Side program) in centering recruiting materials, recruitment efforts and curriculum on valuing equity, diversity and inclusion in the context of neighborhood development. Alumni routinely report either being attracted to the program due to its equity orientation or having their perspectives positively shifted in regard to equitable neighborhood development. Recent scholarship suggests that this orientation of equity as a central value will be more likely to produce more equitable outcomes.

Harwood (2007) argues that Neighborhood Improvement Programs (NIPs) too often focus on non-political, non-confrontational issues such neighborhood aesthetics while sidelining issues of social justice and crime prevention. Harwood case study in Santa Ana, CA notes that “in Santa Ana, the emphasis on cleaning the neighborhood and keeping the ‘politics’ out of neighborhood improvement ultimately depoliticizes many neighborhood activists by limiting the scope of their work and the resources made available to create meaningful social change.” Ultimately, Harwood finds that NIPs can prove hurtful to true progress for communities whose objectives include goals which the city’s bureaucracy has deemed ‘political’ such as social justice, health care, affordable housing, and crime prevention. As an alternative to the top-down NIP approach, Harwood suggests neighborhood-based governance that “gives neighborhoods decision-making power and the resources to promote change without regard to the citizenship of their residents” (Harwood, 2007).

An analysis of the application of community benefit agreements to counter growth machine politics is presented in Colleen Cain’s Negotiating with the Growth Machine. Cain’s case analysis of the aftermath of the sports arena community benefits agreement in Pittsburg, PA finds potential for utilizing community voice to assure some form of “value conscious” growth (e.g. growth that supports community’s needs over capital). Although, Cain’s analysis of the Pittsburg CBA is pessimistic of the long-term potential of CBA’s in this role, noting that CBA’s do not fundamentally alter the political influence and dominant power of growth machine regimes (Cain, 2014). Cain call for a “larger deconstruction” of the growth machine to alter the political and economic domination of growth machine regimes (Cain, 2014, pg. 955).

We argue that the NLA presents a potential model for an equity centered initiative that could contribute to this “larger deconstruction” of the growth machine. As described by the South Side NLA director, communicating values and norms centered on equity and inclusion must be a central part of the NLA and broader community engagement.

*“What if there was sort of a welcome packet for every single new resident and then every single new business that moved in saying, welcome, we're glad you're here? We want new people moving into our community. Here's the resources in your community and here's the history of the South Side who has been here (historically) and our identity across (time). Here are key community values. We are open to everyone. We like diversity. We like the broad range of who we are as a community. And oh, by the way, in our community we don't call the police as a first line of defense. (These) are our values and so welcome, we're glad you're here. But let us define for you what it means to live and do business here.”*

### Channeling Awareness and Anxiety about Gentrification into Equitable Community Change

Programs like the NLA can provide an outlet to capitalize on more privileged recent residents who want to support the community and not gentrify it. As articulated by a NLA director, the program has seen an increase in recent newcomers who are White and economically privileged. These individuals represent an influx of newcomers who were attracted to the neighborhood for its diversity but recognize that they may be part of a gentrification process which could produce displacement.

*“In the last like two, maybe three years of the academy. I've noticed an increase in young white middle class people that have recently moved to the neighborhood and understand that they are the face of gentrification. (They) want to figure out what to do about that. How do they respond to that (issue), maybe they have a little bit of guilt? But recognizing it and wanting to work against the economic pressures.”*

The anxiety around contributing to gentrification and desiring to be a positive force in the neighborhood was commonly referenced by White economically advantaged NLA alumni. As articulated by an NLA alumni below.

*“But we bought our house for $300,000 and the people next door to me. You know, I've been in their house for since the 70’s and I am really terrified of being the person who causes the problem. But at the same time, I want to be part of the solution and somebody was going to buy my house, somebody was going to live here. So, I don't know if it's guilt. I don't think it's guilt, I just I want to be a positive influence. I don't want to just move in here. I’ve, you know, bought the most expensive house on the block. There are plenty of people who are probably at risk of (displacement). Taxes are increasing. Are they going to get pushed out? And so, my head is spinning and is like, I want to be part of this. But I also want to be aware of what the problems are with the changes that are happening in the neighborhood. What I can do to advocate for, you know, positive things to happen. Not just, you know, pure gentrification.”*

Programs like the NLA could potentially leverage this demographic to build a broader base of support for assuring the community remains affordable, accessible and diverse. More importantly, the bridging social capital developed in the program could build a political base to support a different form of community change. A model of neighborhood redevelopment that is truly focused on equity and values diversity as a critical community asset.

#### Centering Equity and Decentering Whiteness and Privilege

Goetz et al. (2020) document the exclusion, value, durability and invisibility of Whiteness in city planning. The traditional process of urban gentrification is built upon Whiteness and centered around a form of racialized capitalism (Burns & Berbary, 2020). An influx of White wealthy homebuyers extracts profits from neighborhoods that had been intentionally devalued through a legacy of redlining and exclusion. Hightower and Fraser (2020) describe this phenomenon as a form of exploitative ‘reverse blockbusting.’ Traditional residents, who are primarily people of color, are displaced and the existence of lower income people in the neighborhood is problematized or implicitly and explicitly associated with a variety of community deficits such as crime or blight (Goetz et al., 2020). The process of gentrification extends beyond the housing market but has implications for increased policing and harassment of people of color (Ramírez, 2020).

The equity centered curriculum of the NLA presents an opportunity to increase the visibility of Whiteness (or surfacing Whiteness) in urban redevelopment. The NLA intentionally targets incoming White affluent residents to engage and expose implicit and explicit biases and to shift perspectives. As described by an NLA program director.

*“We definitely see the south side neighborhood Leadership Academy as one way of instilling that value (racial equity) in the community. And so in that sense it is the white middle class folks that is a target audience. That person that isn't quite sure if Black Lives Matter or isn't quite sure how they feel about like a line (of hard living people) in front of the Free Store. That's the person that I want in this class because that's who I'm trying to reach.”*

As described by an NLA alumni who is a long time South Side resident, his role in the program and as an alumnus is to bring authenticity to engagement and decision making because of his experience.

*“We’ve been that family -- poor, hustling, on public assistance. There were many times that we got the wagon and the backpack and went to the food pantry. I am of this community” *(Price, 2017).

The NLA’s structure challenges the norms of Whiteness in changing urban space and seeks to de-center White privilege in community processes. For example, direct engagement with more marginalized residents enhanced knowledge of the harmful impacts of gentrification. Interviews with program directors and alumni repeatedly identified examples of more privileged academy members learning, reflecting and shifting their behaviors and perspectives due to the intentional engagement of diverse perspectives in the program.

*“Well, while the middle-class person wrote into the grant things like tents and sleeping bags. It was the homeless person that was able to push back on that and say, so I'm homeless, but that doesn't necessarily mean I'm living under a bridge. I'm couch surfing at a friend's house and so a tent is not only useless to me. It's actually a liability.”*

*“(My) whole focus was on a home ownership and just building a sense of community, my whole goal was like to get more people to be homeowners because I feel like when you're a homeowner you're vested in the community. But through (the NLA) what I've discovered...is that you got to meet people where they are, and a lot of folks are not ready to be homeowners and you still need to value them.”*

*“And for me, learning to see and hear people. It's something I'm still working on because I love to talk. I'm still learning not to talk and to listen has been probably one of the more valuable things. I feel like they did that throughout all of the training. There was always an element about learning about your neighbors and not pushing on to your neighbors what you think they need but asking them what they need.”*

### Structural Factors Contributing to Program Sustainability

Only the South Side NLA is still active and has sustained operations throughout the challenges of the COVID-19 pandemic. Although the United Way is no longer funding the South Side NLA, the program is now funded internally by the local CDC (CD4AP). The organization housing the NLA on the East Side went through a leadership transition and reorganization. The Linden NLA was temporarily based out of a nearby Settlement house but never found a strong local organization to host the academy. In contrast, CD4AP was deeply engaged in the design of the South Side NLA and has had stability throughout the NLA’s six years of existence. Thus, sustainability for programs like the NLA requires a robust community organization whole values align with the academy and can provide long term stability.

The alignment of more structural interventions must compliment grass roots leadership development. Amplifying the effectiveness of the South Side NLA are structural conditions which have fostered equitable development activities. The South Side neighborhood is home to the largest hospital based affordable housing initiative in the United States, the Healthy Neighborhood Healthy Families initiative (Kelleher et al., 2018). The collaborative program between Nationwide Childrens Hospital, Community Development for All People and other nonprofit, for profit and public sector partners has invested more than $70 million (USD) in affordable housing in the South Side community for the past thirteen years. Additional investments in earlier childhood development, workforce development, youth development and food security have complimented the large investment in affordable housing. The durability and impact of the South Side NLA cannot be disentangled from the larger structural investments in supporting diversity and inclusion in the neighborhood.

### The Neoliberal Era: The Role of Philanthropy in Cultivating Community Power

The need to foster grassroots leadership in the neoliberal era is paramount. During the 1980s, there was a withdrawal and reduction of federal programs that forced local government to generate revenue in ways that exacerbated intermunicipal competition for businesses, capital and middle-class residents (Eisinger, 1998; Hackworth, 2007). Our findings suggest that locally driven efforts to cultivate grass roots community leaders, are even more vital in the contemporary neoliberal era. Arnstein’s Ladder was developed during a time period of substantial government interventions in urban development (Arnstein, 1969). In the following half century since its release, cities have experienced devolution and a shrinking federal role in funding urban development. In the era of the entrepreneurial city, neighborhoods can no longer rely upon federal funding, leaving redeveloping neighborhoods particularly vulnerable to market driven gentrification and displacement. In this void of resources, philanthropic efforts to support robust grass roots leadership is the last remaining defense against widespread displacement and the primary asset to support equitable development practices.

We must also recognize that philanthropic efforts to counter gentrification pressures and support equitable development involves more than just financial investment. In the case of the South Side neighborhood leadership academy, the United Way was not only a financial supporter, but was essential as a convener to bring together multiple organizations and stakeholders who were philosophically aligned (Community Development for All People, Nationwide Childrens Hospital, the Kirwan Institute) and seeking to address a common challenge in the South Side neighborhood (displacement). The various institutional partners co-developed a program and curriculum was equity focused and centered neighborhood inclusion and diversity as a primary value. In this case, the foundation provided not only direct investment and convening, but also utilized its internal expertise (guiding participants in understanding grant writing and fund raising for local projects) and by leveraging the expertise of its extensive local networks of stakeholders to leadership academy students and alumni.

## Appendix

**Addendum: Survey and Interview Instruments**

***Interview Questions for Alumni***

Motivations and Experience:

1. What motivated you to join the leadership academy?
2. The academy usually has each graduate work on a community project, what were your projects in the leadership academy?
3. What type of issues do you work on in your community or neighborhood?
4. Do you feel more of a connection to your neighborhood and city as after graduating from the leadership academy?
  - Can you elaborate on how you feel more connected?
5. What subject material had the deepest impact on you from the leadership academy curriculum (select all that apply)?
6. Do you think the academy prepared you to be a leader (for example, to implement leadership skills) in your community? If so, how? If not, why?

Perceptions of Community Change:

7. Do you think that the leadership academy made you more aware about the needs and issues that are important to your neighborhood/community?
8. Has the leadership academy influenced your perspective, views or knowledge about neighborhood change, neighborhood redevelopment or concerns about displacement of your neighbors?
9. Do you feel more aware about what is going on in your neighborhood/community after graduating? i.e. development projects, community events, issues that the community is facing
10. In what ways has your experience through the leadership academy changed how you see the future of your neighborhood (are you more optimistic or less optimistic)?

Agency and Empowerment:

11. Do you feel more prepared to be involved in government meetings and express your opinions and concerns at these meetings or to engage and advocate with decision makers?
12. Do you feel that the leadership academy prepared you to have the ability to organize members of your community to speak out on issues that are important to your community?
13. Has your experience in the leadership academy made you feel more empowered to influence the future of your neighborhood?
14. After participating in the academy do you feel more optimistic that community input can create change in your neighborhood and city?
15. Do you feel leadership academy leaders in your neighborhood can work to assure neighborhoods change in a way that benefits all residents, particularly those who are more vulnerable or marginalized?
16. Do you feel leadership academy leaders in your neighborhood can work to help assure more vulnerable or marginalized residents are not displaced as the neighborhood changes?

Networks and Organizing:

17. Do you stay in contact with other academy graduates since graduating from the leadership academy?
  - If Yes, how many graduates do you stay in contact with?
18. How many organizations, initiatives or programs have you become involved in since graduating from the leadership academy?
19. How much time do you contribute to community work since graduating from the leadership academy (hours a week or hours a month)?
20. Have you played a leadership role in any community meetings or engagements since graduating from the academy? (i.e. served on task force, area commission, civic association, government citizens committee)
21. In what ways do you think the leadership academy can be strengthened?

***Interview Questions for Program Directors or Staff***

Motivations and Experience:

1. What do you see motivating most of the applicants to the leadership academy?
2. What are some of the more memorable projects that academy graduates worked on in the community? What kinds of issues do the graduates tend to focus on?
3. What subject material in the curriculum has the deepest impact on participants?
4. Do you feel that the graduates feel a deeper connection to the neighborhood after graduating from the leadership academy?
5. What does the diversity of leadership academy alumni look like?
6. How do you work in issues of equity, diversity and inclusion into the NLA structure and curriculum?

Perceptions of Community Change:

7. Do you think that the leadership academy makes alumni more aware about the needs and issues that are important to your neighborhood/community?
8. Do you feel the leadership academy influenced the perspective, views or knowledge of participants about neighborhood change, neighborhood redevelopment or concerns about displacement of your neighbors?
9. In what ways has your experience with leadership academy alumni changed how you see the future of your neighborhood (are you more optimistic or less optimistic)?

Agency and Empowerment:

10. Do you feel the leadership academy makes graduates feel more empowered to influence the future of the neighborhood?
11. Do you feel like the alumni will be able to create change in the neighborhood?
12. Do you feel leadership academy leaders in the neighborhood can or are working to assure neighborhoods change in a way that benefits all residents, particularly those who are more vulnerable or marginalized?
13. Do you feel leadership academy leaders in the neighborhood can or are working to help assure more vulnerable or marginalized residents are not displaced as the neighborhood changes?

Networks and Organizing:

14. Do you stay in contact with academy alumni after they graduate from the leadership academy?
15. Do you see alumni working with each other after they graduate?
16. Do you see alumni active in organizations, initiatives or programs since graduating from the leadership academy?
17. In what ways do you think the leadership academy can be strengthened?
18. For other neighborhoods that are experiencing neighborhood change, do you think a program like the NLA would be beneficial in supporting the needs of marginalized communities and avoiding neighborhood displacement?

***Survey Questions for Alumni***

19. What were your projects in the leadership academy? (Please mark as N/A if you did not lead a community project as part of your NLA experience).
  - Please Describe: Text Box for Answer
20. What type of issues do you work on in your community or neighborhood (select all that apply)?
  - Education
  - Safety
  - Health
  - Housing
  - Community Development
  - Employment
  - Environment
  - Arts/Culture
  - Transportation
  - Social Services
  - Other:
21. How long has it been since you graduated from the leadership academy?
  - Six months or less
  - Six months to 12 months
  - 12 months to 24 months
  - 24 months or longer
22. Which leadership academy did you graduate from?
  - East Side
  - Linden
  - South Side
  - Central (downtown)
23. Do you feel more of a connection to your neighborhood and city as after graduating from the leadership academy?
  i. Yes
  ii. NoUnsure
    b. If you answered yes, can you elaborate on how you feel more connected?
      i. Text Box for Answer
24. What subject material had the deepest impact on you from the leadership academy curriculum (select all that apply)?
  - Personal Development/Team Building
  - Inclusive Leadership
  - Community Engagement
  - Strategic Planning
  - Fundraising/Grant Writing
  - Networking/Coalition Building
  - Community Development Tools (for example, learning about asset based community building)
  - Political Advocacy Tools
  - Local (neighborhood) Knowledge
  - Diverse neighborhood knowledge – Central Community Tour
  - International neighborhood knowledge – Central Community Tour
  - Understanding media
  - Understanding diversity
  - Other (please describe): text box
25. Do you think the academy prepared you to be a leader (for example, to implement leadership skills) in your community? If so, how? If not, why?
  - Yes
  - No
  - Unsure
  - If you answered yes, how did the academy prepare you to be a leader? (Text Box)
26. Do you think that the leadership academy made you more aware about the needs and issues that are important to your neighborhood/community?
  - Much more aware
  - More aware
  - No Change
  - Less aware
  - Much less aware
27. How many organizations, initiatives or programs have you become involved in since graduating from the leadership academy?
  - None
  - 1
  - 2 to 4
  - More than 4
  - What organizations or initiatives: (please list)
28. How much time do you contribute to community work since graduating from the leadership academy?
  - Less than 1 h a week
  - 1 to 4 h a week
  - 4 to 10 h a week
  - 10 to 20 h a week
  - 20 or more hours a week
29. Do you feel more aware about what is going on in your neighborhood/community after graduating? i.e. development projects, community events, issues that the community is facing
  - Much more aware
  - More aware
  - No Change
  - Less aware
  - Much less aware
30. Do you feel more prepared to be involved in government meetings and express your opinions and concerns at these meetings or to engage and advocate with decision makers?
  - Much more prepared
  - More prepared
  - No Change
  - Less prepared
  - Much less prepared
31. (How many instances have you been involved with local or state government meetings?)
  - Text box
32. In what ways has your experience through the leadership academy changed how you see the future of your neighborhood?
  - Much more optimistic
  - More optimistic
  - No change
  - Less optimistic
  - Much less optimistic
  - Other comments: (text box)
33. Has your experience in the leadership academy made you feel more empowered to influence the future of your neighborhood?
  - I feel much more empowered
  - I feel more empowered
  - No change
  - I feel less empowered
  - I feel much less empowered
  - Other comments: (Text Box)
34. Do you stay in contact with other academy graduates since graduating from the leadership academy?
  - Yes
  - No
  - If Yes, how many graduates do you stay in contact with?
35. How many community meetings or engagements have you gone to since graduating the academy?
  - Less than 2
  - 2 to 5
  - 5 to 10
  - 10 to 20
  - 20 or more
36. Have you played a leadership role in any community meetings or engagements since graduating from the academy? (i.e. served on task force, area commission, civic association, government citizens committee)
  - Yes
  - No
  - If Yes, can you describe (Text Box)
37. Do you feel that neighbors or other communities see you as a resource for news, assistance or leadership in reference to what is happening in the neighborhood and/or the city?
  - Yes
  - No
  - Unsure
  - If you answered yes, can you provide some examples: (text box)
38. Since you attended the academy do you work to to share news about what is going on in your neighborhood and/or city?
  - Yes
  - No
  - If Yes, how do you engage the community? (Text Box)
  - What medium do you use to share information?
    i. Social media
    ii. Newsletters,
    iii. Attending meetings
    iv. Personal meeting with neighborhood stakeholders
    v. Other
39. Do you feel that the leadership academy prepared you to have the ability to organize members of your community to speak out on issues that are important to your community?
  - Much more prepared
  - More prepared
  - No change
  - Less prepared
  - Much less prepared
  - Other comments: (text box)
40. After participating in the academy do you feel more optimistic that community input can create change in your neighborhood and city?
  - Much more optimistic
  - More optimistic
  - No change
  - Less optimistic
  - Much less optimistic
  - Other comments: (text box)
41. Do you feel leadership academy leaders in your neighborhood can work to assure neighborhoods change in a way that benefits all residents, particularly those who are more vulnerable or marginalized?
  - Strong agree
  - Agree
  - Unsure
  - Disagree
  - Strongly disagree
  - Other comments: (text box)
42. Do you feel leadership academy leaders in your neighborhood can work to help assure more vulnerable or marginalized residents are not displaced as the neighborhood changes?
  - Strong agree
  - Agree
  - Unsure
  - Disagree
  - Strongly disagree
  - Other comments: (text box)
43. In what ways do you think the leadership academy can be strengthened? Text Box
44. Do you feel that networking with other NLA alumni is beneficial or have you networked with other NLA for problem solving? (Text Box)
